# Performance of ChatGPT and Microsoft Copilot in Bing in answering obstetric ultrasound questions and analyzing obstetric ultrasound reports

**DOI:** 10.1038/s41598-025-99268-2

**Published:** 2025-04-26

**Authors:** Yanran Du, Chao Ji, Jiale Xu, Minyan Wei, Yunyun Ren, Shujun Xia, JianQiao Zhou

**Affiliations:** 1 Department of Ultrasound, Ruijin Hospital, Shanghai Jiaotong University School of Medicine, No. 197, Rui Jin 2nd Road, Shanghai, 200025 China; 2 Department of Pediatrics, Ruijin Hospital, Shanghai Jiaotong University School of Medicine, No. 197, Rui Jin 2nd Road, Shanghai, 200025 China; 3 Obstetrics and Gynecology Hospital of Fudan University, No.128, Shenyang Road, Shanghai, 200090 China

**Keywords:** Large language models, ChatGPT, Microsoft Copilot in Bing, Obstetric ultrasound, Health care, Ultrasonography

## Abstract

To evaluate and compare the performance of publicly available ChatGPT-3.5, ChatGPT-4.0 and Microsoft Copilot in Bing (Copilot) in answering obstetric ultrasound questions and analyzing obstetric ultrasound reports. Twenty questions related to obstetric ultrasound were answered and 110 obstetric ultrasound reports were analyzed by ChatGPT-3.5, ChatGPT-4.0 and Copilot, with each question and report being posed three times to them at different times. The accuracy and consistency of each response to twenty questions and each analysis result in the report were evaluated and compared. In answering twenty questions, both ChatGPT-3.5 and ChatGPT-4.0 outperformed Copilot in accuracy (95.0% vs. 80.0%) and consistency (90.0% and 85.0% vs. 75.0%). However, no statistical difference was found among them. When analyzing obstetric ultrasound reports, ChatGPT-3.5 and ChatGPT-4.0 demonstrated superior accuracy compared to Copilot (*P* < 0.05), and all three showed high consistency and the ability to provide recommendations. The overall accuracy and consistency of ChatGPT-3.5, ChatGPT-4.0, and Copilot were 83.86%, 84.13% vs. 77.51% in accuracy, and 87.30%, 93.65% vs. 90.48% in consistency, respectively. These large language models (ChatGPT-3.5, ChatGPT-4.0 and Copilot) have the potential to assist clinical workflows by enhancing patient education and patient clinical communication around common obstetric ultrasound issues. With inconsistent and sometimes inaccurate responses, along with cybersecurity concerns, physician supervision is crucial in the use of these models.

**Supplementary Information:**

The online version contains supplementary material available at 10.1038/s41598-025-99268-2.

## Introduction

Large language models (LLMs) are artificial intelligence (AI) systems that are trained on billions of words derived from articles, books and other internet-based content, can respond to free-text queries without being specifically trained in the task in question, causing excitement and concern about their use in healthcare settings^1^. Nowadays, individuals often turn to the Internet or communicate with their physicians to obtain healthcare information. In November 2022 and March 2023, OpenAI released ChatGPT-3.5 and its upgraded version, ChatGPT-4.0, respectively^2,3^. In May 2023, Microsoft launched Copilot in Bing (currently Copilot)^4^. These tools are available to the public through free online platforms accessible to all users with free accounts. Using a chat-based interface, these AI models can provide well-formulated, conversational, easy-to-understand and comprehensive responses. All three are commonly used LLMs that can use converters to generate text that is very similar to human language.

Many studies have been conducted to evaluate the applicability of ChatGPT and Copilot in solving medical related problems. ChatGPT has been shown to be appropriate in answering cardiovascular disease prevention-related questions, accurate in answering common cirrhosis and hepatocellular carcinoma-related questions and great potential to automate provision of accurate healthcare information related to breast cancer prevention and screening^5–7^. It can also transform free-text thyroid ultrasound reports into structured formats in the ultrasound field^8^. Subsequent studies have compared the performance of ChatGPT and Bing Chat across various medical domains. Though the performance of different models can vary greatly, their potential use as a clinical tool is easy to see because it has demonstrated the ability to accurately and repetitively answer clinical questions in clear and concise language, which can be understood by healthcare providers and patients^9–11^. At the same time, the application of AI models in the medical field remains a subject of significant debate and controversy^12–15^. Researches have shown that recognizing its limitations is crucial, as seemingly satisfactory answers may be misleading without appropriate caution.

To further evaluate and compare the current capabilities of LLMs in the field of obstetric ultrasound. We designed twenty questions related to obstetric ultrasound examination and included 110 obstetric ultrasound reports in this study. The accuracy and consistency of responses provided by ChatGPT-3.5, ChatGPT-4.0 and Copilot to obstetric ultrasound questions and the analysis of report findings were evaluated and compared.

## Results

### Analysis results of twenty questions related to obstetric ultrasound

ChatGPT-3.5, ChatGPT-4.0 and Copilot answered all questions. The comparison of consistency and accuracy among ChatGPT-3.5, ChatGPT-4.0 and Copilot in three responses to each question is shown in Table 1. Among the responses to 20 questions, those generated by ChatGPT-3.5 were consistent for 18, ChatGPT-4.0 for 17, and Copilot for 15.

**Table 1:** Comparison of consistency and accuracy among ChatGPT-3.5, ChatGPT-4.0 and Microsoft copilot in Bing in three responses to each question.

	Responses generated by			*P* value
	Microsoft Copilot in Bing	ChatGPT-3.5	ChatGPT-4.0	
Consistency				*P*_*1*_ = 0.212, *P*_*2*_ = 0.429, *P*_*3*_ *=* 0.633
Consistent	75.00%(15/20)	90.00%(18/20)	85.00%(17/20)	
Inconsistent	25.00%(5/20)	10.00%(2/20)	15.00%(3/20)	
Accuracy				*P*_*1*_ = 0.151, *P*_*2*_ = 0.151, *P*_*3*_ > 0.999
1. Comprehensive	40.00%(8/20)	75.00%(15/20)	75.00%(15/20)	
2. Correct but inadequate	40.00%(8/20)	20.00%(4/20)	20.00%(4/20)	
3. Some correct and some incorrect	20.00%(4/20)	5.00%(1/20)	5.00%(1/20)	
4. Completely incorrect	0.00%(0/20)	0.00%(0/20)	0.00%(0/20)	

*P*_*1*_, *P*_*2*_, and *P*_*3*_ represent the *P*-values between Copilot and ChatGPT-3.5, Copilot and ChatGPT-4.0, and between ChatGPT-3.5 and ChatGPT-4.0, respectively. There is no significant difference in the consistency and accuracy of responses to each question among Copilot, ChatGPT-3.5, and ChatGPT-4.0 (*P* > 0.05 for all).

Considering the accuracy and completeness of the responses, 95.0% (19/20) of the responses generated by ChatGPT-3.5 and ChatGPT-4.0 were correct, and 75.00% (15/20) of them were very comprehensive. However, in the responses generated by Copilot, 80.00%(16/20) of the answers were correct, and only 40%(8/20) of them were very comprehensive. For example, Copilot’s response on ultrasound frequency in Trial 2 (Table S1), ChatGPT-3.5’s response on amniotic fluid index in Trial 3(Tables S2), and ChatGPT-4.0’s response on placental maturity level II in Trial 2 (Tables S3) are all incorrect.

Some responses to some questions were correct but inadequate. One of ChatGPT-3.5’s responses to question 13 about the explanation of the distance between the lower margin of the placenta and the internal cervical orifice orificium internum uteri was some correct and some incorrect, of which “low-lying placenta” was not mentioned and gestational age was not taken into account. Regarding the explanation of biparietal diameter/head circumference/abdominal circumference/femur and humeral length less than − 2SD, some responses to question 7 lacked relevant details, including maternal factors, placental factors, fetal factors, and genetic factors. No responses were graded as completely incorrect.

There was no significant difference among ChatGPT-3.5, ChatGPT-4.0 and Copilot in the consistency and accuracy of the responses to each question (*P* > 0.05 for all).

### Interpretation results of ultrasound reports

#### Population

In this study, ChatGPT-3.5, ChatGPT-4.0 and Copilot generated report analysis results from 110 ultrasound reports of 107 pregnant women. The mean ± SD age of the pregnant women was 31 ± 3.76 years. The characteristics of the study cohort are summarized in Table 2.

**Table 2:** Basic characteristics of patients.

Characteristics	*n* (%)
Cases	110
Maternal age (years)	31 ± 3.76
Gestational age at ultrasound	
28 + 0 to 36 + 6weeks	28 (25.45%)
37 + 0 to 38 + 6weeks	38 (34.55%)
≥ 39 + 0 weeks	44 (40.00%)
Mode of delivery	
Spontaneous vaginal	67 (60.91%)
Cesarean	43 (39.09%)
Birth weight (g)	3211 ± 511
Sex of newborn	
Female	59 (53.64%)
Male	51 (46.36%)
5-min Apgar score	
≤ 7	3 (2.73%)
> 7	107 (97.27%)
Neonatal prognosis	
Normal	107(97.27%)
Abnormal^#^	3 (2.73%)

^#^ Three of the newborns have abnormal prognoses. One newborn has been diagnosed with neonatal respiratory distress syndrome, extremely low birth weight, and neonatal infection. The second newborn has been diagnosed with transient tachypnea of the newborn, neonatal hyperbilirubinemia, and small for gestational age. The third newborn has been diagnosed with pneumonia, congenital thrombocytopenia, and neonatal hyperbilirubinemia.

According to the presence of abnormal indicators, obstetric ultrasound reports could be classified as follows:

One abnormal indicator: 17 cases (15.45%) of abnormal fetal growth measurements, 18 cases (16.36%) of abnormal amniotic fluid index, and 6 cases (5.45%) of abnormal placental findings.

Two abnormal indicators: 13 cases (11.82%) of both abnormal fetal growth measurements and amniotic fluid index, and 1 case (0.91%) of both abnormal placental findings and fetal growth measurements.

Three abnormal indicators: 1 case (0.91%) with abnormal placental findings, fetal growth measurements, and amniotic fluid index.

Completely normal: 54 cases (49.09%).

#### **Quality of analyzed ultrasound reports**

Each report was input three times separately into ChatGPT-3.5, ChatGPT-4.0 and Copilot software. Given that there were 110 reports, 990 analysis results were generated. If a report conclusion contained two or more abnormal indicators, each abnormality was evaluated individually. For example, when an ultrasound report includes both abnormal fetal growth measurements and amniotic fluid index, each abnormality will be counted as a separate analysis result. Therefore, a total of 1,134 ultrasound report analysis results were obtained, among which 288 cases with abnormal fetal growth measurements, 288 cases with abnormal amniotic fluid index, 72 cases with abnormal placental findings and 486 cases with normal results.

The comparison of the accuracy of ChatGPT-3.5, ChatGPT-4.0 and Copilot analysis ultrasound reports was shown in Table 3. Overall, ChatGPT-3.5 and ChatGPT-4.0 demonstrated superior accuracy in analyzing ultrasound reports compared to Copilot, with no statistically significant difference in accuracy between the two (*P*_ChatGPT−3.5 vs. Copilot_=0.027, *P*_ChatGPT−4.0 vs. Copilot_=0.021, *P*_ChatGPT−3.5 vs. ChatGPT−4.0_=0.921). In the results generated by ChatGPT-3.5, 83.86% (317 of 378) were correct and 16.14% (61 of 378) were incorrect; for ChatGPT-4.0, 84.13% (318 of 378) were correct and 15.87% (60 of 378) were incorrect; and for Copilot, 77.51% (293 of 378) were correct and 22.49% (85 of 378) were incorrect. However, when analyzing cases of abnormal fetal growth measurements, abnormal placental findings, and normal results, the accuracy of responses from all three software programs was similar (*P* > 0.05). Only in the cases of abnormal AFI did ChatGPT-3.5 and ChatGPT-4.0 demonstrate higher accuracy than Copilot(*P* < 0.05). The accuracy of the three software in analyzing abnormal fetal growth measurements was relatively low: ChatGPT-3.5 at 59.38%, ChatGPT-4.0 at 60.42%, and Copilot at 50.00%. Among the three analyses of the same ultrasound report, ChatGPT-3.5, ChatGPT-4.0 and Copilot all showed high consistency (Table 4). Additionally, each analysis report provides detailed recommendations and feedback. Examples of ultrasound report analysis results were shown in Table S4.

**Table 3:** Comparison of the accuracy among ChatGPT-3.5, ChatGPT-4.0 and Microsoft copilot in Bing in analysis of ultrasound reports.

	ChatGPT-3.5	ChatGPT-4.0	Microsoft Copilot in Bing	*P* value
Abnormal fetal growth measurements				*P*_*1*_ = 0.192,*P*_*2*_ = 0.147, *P*_*3*_ = 0.883
Accurate	57(57/96,59.38%)	58(58/96,60.42%)	48(48/96,50.00%)	
Inaccurate	39(39/96,40.62%)	38(38/96,39.58%)	48(48/96,50.00%)	
Abnormal AFI				*P*_*1*_ = 0.001^*^,*P*_*2*_ = 0.021^*^, *P*_*3*_ = 0.233
Accurate	84(84/96,87.50%)	78(78/96,81.25%)	64(64/96,66.67%)	
Inaccurate	12(12/96,12.50%)	18(18/96,18.75%)	32(32/96,33.33%)	
Abnormal placental findings				*P*_*1*_ = 0.489,*P*_*2*_ = 1.000, *P*_*3*_ = 0.489
Accurate	22(22/24,91.67%)	24(24/24,100.00%)	24(24/24,100.00%)	
Inaccurate	2(2/24,8.33%)	0(0/24,0.00%)	0(0/24,0.00%)	
Normal results				*P*_*1*_ = 0.396,*P*_*2*_ = 0.735, *P*_*3*_ = 0.239
Accurate	154(154/162,95.06%)	158(158/162,97.53%)	157(157/162,96.91%)	
Inaccurate	8(8/162,4.94%)	4(4/162,2.47%)	5(5/162,3.09%)	
All				*P*_*1*_ = 0.027^*^,*P*_*2*_ = 0.021^*^, *P*_*3*_ = 0.921
Accurate	317(317/378,83.86%)	318(318/378,84.13%)	293(293/378,77.51%)	
Inaccurate	61(61/378,16.14%)	60(60/378,15.87%)	85(85/378,22.49%)	

*P*_*1*_, *P*_*2*_, and *P*_*3*_ represent the *P*-values between Copilot and ChatGPT-3.5, Copilot and ChatGPT-4.0, and between ChatGPT-3.5 and ChatGPT-4.0, respectively. ^*^ *P* < 0.05 was considered statistically significant. AFI: Amniotic Fluid Index.

**Table 4:** Comparison of the consistency among ChatGPT-3.5, ChatGPT-4.0 and Microsoft copilot in Bing in analysis of ultrasound reports.

	ChatGPT-3.5	ChatGPT-4.0	Microsoft Copilot in Bing	*P* value
Abnormal fetal growth measurements				*P*_*1*_ = 1.000,*P*_*2*_ = 0.613, *P*_*3*_ = 0.613
Consistent	29(29/32,90.63%)	31(31/32, 96.87%)	29(29/32,90.63%)	
Inconsistent	3(3/32,9.37%)	1(1/32,3.13%)	3(3/32,9/37%)	
Abnormal AFI				*P*_*1*_,*P*_*2*_ = 1.000, *P*_*3*_ = 0.740
Consistent	26(26/32,81.25%)	27(27/32,84.38%)	27(27/32,84.38%)	
Inconsistent	6(6/32,18.75%)	5(5/32,15.63%)	5(5/32,15.63%)	
Abnormal placental findings				*P*_*1*_,*P*_*3*_ = 0.467, *P*_*2*_ = 1.000
Consistent	6(6/8,75.00%)	8(8/8,100.00%)	8(8/8,100.00%)	
Inconsistent	2(2/8,25.00%)	0(0/8,0.00%)	0(0/8,0.00%)	
Normal results				*P*_*1*_ = 1.000,*P*_*2*_ = 0.678, *P*_*3*_ = 0.437
Consistent	49(49/54,90.74%)	52(52/54,96.30%)	50(50/54,92.59%)	
Inconsistent	5(5/54,9.26%)	2(2/54,3.70%)	4(4/54,7.41%)	
All				*P*_*1*_ = 0.423,*P*_*2*_ = 0.351, *P*_*3*_ = 0.086
Consistent	110(110/126,87.30%)	118(118/126,93.65%)	114(114/126,90.48%)	
Inconsistent	16(16/126,12.70%)	8(8/126,6.35%)	12(12/126,9.52%)	

*P*_*1*_, *P*_*2*_, and *P*_*3*_ represent the *P*-values between Copilot and ChatGPT-3.5, Copilot and ChatGPT-4.0, and between ChatGPT-3.5 and ChatGPT-4.0, respectively. There is no significant difference in the consistency of responses to each question among Copilot, ChatGPT-3.5, and ChatGPT-4.0 (*P* > 0.05 for all).

AFI: Amniotic Fluid Index.

## Discussion

This study evaluated the accuracy and consistency of AI models (ChatGPT-3.5, ChatGPT-4.0, and Copilot) in answering obstetric ultrasound questions and analyzing obstetric ultrasound reports. ChatGPT-3.5 and ChatGPT-4.0 demonstrated superior accuracy and consistency in answering 20 ultrasound-related questions compared to Copilot. However, there was no statistical difference among the models (*P* > 0.05 for all), which may be due to the small sample size. In the analysis and interpretation of obstetric ultrasound reports, both ChatGPT-3.5 and ChatGPT-4.0 exhibited significantly higher accuracy than Copilot (*P* < 0.05), with all models showing high consistency.

These advanced language models have demonstrated potential as clinical aids, offering clear and typically accurate responses to medical questions that are understandable by both healthcare providers and patients. While observing its capability, it is important to characterize its limitations^16^. In this study, ChatGPT-3.5, ChatGPT-4.0 and Copilot may provide inconsistent or not entirely correct responses. For example, Copilot’s responses to placental maturity and frequency of ultrasound examination during pregnancy, ChatGPT-3.5’s response to the amniotic fluid index, and ChatGPT-4.0’s response to placental maturity level II, which have been reported three times but using different descriptions and resulted in some correct and some incorrect answers. The final suggestion for many responses generated by these models was to consult with the healthcare provide. It can be found that models similar to ChatGPT-3.5, ChatGPT-4.0 and Copilot can generate coherent and grammatically correct text, but they may not have the ability to distinguish between each patient, combine patient medical history, and combine with the latest advances in this technology, as human experts in specific fields do.

For the analysis of obstetric ultrasound reports, three LLMs were able to identify most abnormal indicators and demonstrate high repeatability. The accuracy of ChatGPT-3.5, ChatGPT-4.0, and Copilot was 83.86%, 84.13%, and 77.51%, respectively, while their consistency was 87.30%, 93.65%, and 90.48%, respectively. However, in identifying abnormalities in fetal growth measurements, three LLMs showed lower accuracy, with ChatGPT-3.5 at 59.38%, ChatGPT-4.0 at 60.42% and Copilot at 50.00%. The study by Rahsepar AA et al.^13^showed that the errors provided by LLMs might be due to their training on diverse internet content such as articles, books, Wikipedia, news, and websites, rather than on scientific literature and information. Another reason might be that the standards for fetal growth measurements vary across different ethnicities^17^, causing the LLMs to reference standards that may not align with the population included in this study. In Shen Y et al.‘s study, it was pointed out that these LLMs do not engage in interactions to clarify the questions being asked to provide accurate answers. Instead, they tend to assume what the user wants to hear, which can result in inaccurate or incomplete information^16^. Furthermore, it is worth noting the security of AI models used in the medical field. If the model is affected by adversarial attacks or erroneous inputs, it can lead to incorrect report interpretation, which may have serious consequences in medical decision-making^18–20^.

In two instances of obstetric ultrasound report analysis, Copilot incorrectly identified a distance greater than 2 cm between the placenta and the cervical os as abnormal, labeling the condition as “placenta previa” and suggesting that it could lead to bleeding during delivery. The dissemination of such incorrect information can cause emotional distress for the pregnant woman and her entire family. A study suggested that we should be cautious about the potential applications of complex natural language processing applications in healthcare^21^. The research results of Ayers JW et al. showed that ChatGPT can provide high-quality and empathetic responses to patient questions raised in online forums, with 78.6% of evaluators preferring chatbot responses^22^. However, Studies have highlighted that it is crucial to recognize that chatbot responses may not always be accurate, as their training datasets may contain biased information, potentially leading to hallucinated responses^13^. These are similar to our research findings, where LLMs were able to analyze each ultrasound report and provide detailed recommendations and opinions, although their analysis results were not entirely accurate.

When comparing the analysis results of ultrasound reports among ChatGPT-3.5, ChatGPT-4.0, and Copilot, it was found that although ChatGPT demonstrated higher overall accuracy than Copilot, each software has its own strengths and weaknesses. The responses generated by ChatGPT-3.5 were more concise and clear, also providing recommendations for each report. The responses generated by ChatGPT-4.0 were very detailed and comprehensive, and a summary was provided at the end of each answer. Copilot’s answers analyzed each item according to the structure of the ultrasound reports, resulting in more detailed and comprehensive final recommendations.

There are several limitations to this study. First, only twenty questions related to obstetric ultrasound were designed and 110 obstetric ultrasound reports were analyzed. Expanding the sample size, especially with samples from different modalities, would better validate the stability and accuracy of the application of LLMs in the medical field. Second, recent advances in decision fusion networks have shown significant success in image classification, especially in multi-modal fusion and multi-task decision-making^23,24^. In this study, only textual data related to obstetric ultrasound were analyzed. Multi-modal data would be explored in future research, which may yield different results. Third, the evaluation of the responses generated by the LLMs was performed by ultrasound doctors with different seniority. Future studies could include obstetricians or maternal-fetal medicine experts, who are highly skilled and proficient in interpreting obstetric ultrasounds and can provide expert clinical guidance.

In summary, these artificial intelligence models (ChatGPT-3.5, ChatGPT-4.0 and Microsoft Copilot in Bing) have the potential to assist clinical workflows by enhancing patient education and patient clinical communication around common obstetric ultrasound issues. However, given the inconsistent and sometimes inaccurate responses, as well as cybersecurity concerns, the supervision of physician is crucial in the use of these models.

## Methods

**Fig. 1:**
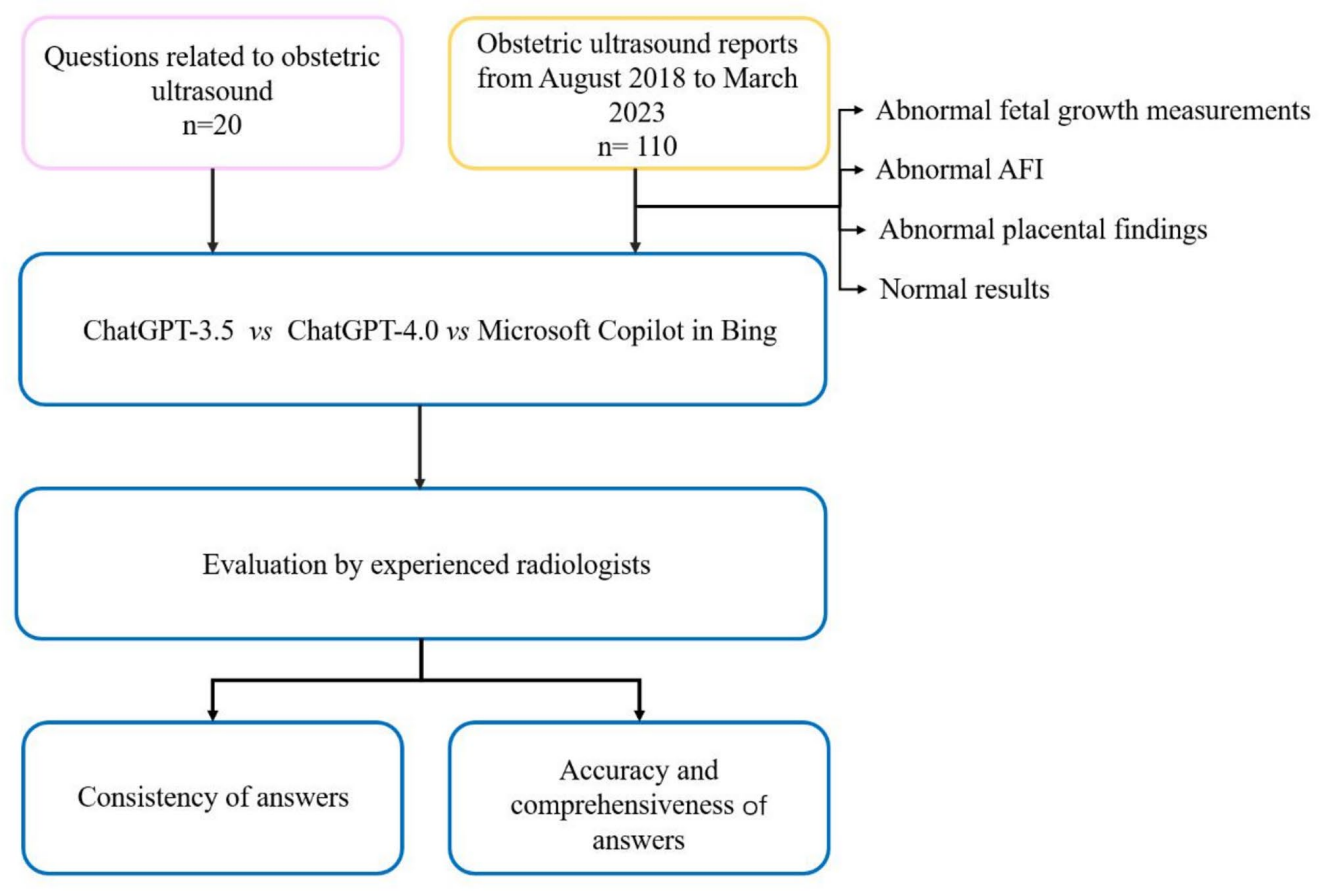
Schematic diagram of the research workflow. AFI: amniotic fluid index.

The flowchart of the study is presented in Fig. 1.

### Questions related to obstetric ultrasound

Twenty questions related to obstetric ultrasound have been created, including the preparation before ultrasound examination, the frequency of ultrasound examination during pregnancy, basic concepts of ultrasound report indicators and the interpretation of ultrasound results, based on the reports of obstetric ultrasound examination and our clinical experience in obstetric ultrasound examination (Table S5). Each question was posed three times to the online ChatGPT-3.5, ChatGPT-4.0 (chat.openai.com) and Microsoft Copilot in Bing (bing.com) at different times, and the responses were recorded. Each set of three responses was graded by two radiologists with two years of experience in obstetric ultrasound(X.J.L., W.M.Y.) and two with ten years of experience in obstetric ultrasound(D.Y.R., X.S.J.), who reached a consensus opinion using publicly available information contained in the guideline of ultrasound in obstetrics^25,26^, the book of diagnostic ultrasound in obstetrics and gynecology^27^, and combined with their clinical experience. Discrepancies in assessment among the radiologists were resolved by a third ultrasound expert (Z.J.Q), who has over twenty years of experience in obstetric ultrasonic diagnosis. The reviewers divided each set of responses into “consistent” or “inconsistent” based on whether the content of the three responses is similar. Then, the accuracy of each response was graded with the following scale: (1) Comprehensive: Defined as accurate and comprehensive, nothing more need to be added by an experienced radiologist; (2) Correct but inadequate: All information is correct but incomplete; Some important information should be added by an experienced radiologist; (3) Some correct and some incorrect; (4) Completely incorrect. If the responses were consistent, only the first responses from ChatGPT-3.5, ChatGPT-4.0 and Copilot were graded. If the responses were inconsistent, all the responses were graded individually. Among the three answers to each question, as long as one answer is grated as incomplete or incorrect, the final accuracy of this question would be graded as incomplete or incorrect.

### Interpretation of ultrasound reports

#### Patients

From August 2018 to March 2023, there were 110 obstetric ultrasound reports from 107 pregnant women with singleton pregnancy selected in this study, at gestational ages ranging from 32.0 to 41.2weeks. Gestational age was determined by last menstrual period and verified by first-trimester dating ultrasound (crown–rump length). All participating women included in the study gave written informed consent for the use of ultrasound reports and clinical data. All the methods hereby explained were performed in accordance with the relevant guidelines and regulations and approved, together with the study protocol, by the ethics committee of the Obstetrics and Gynecology Hospital Affiliated to Fudan University (2018-73). The enrolment criteria for the study were: (1) singleton pregnancy; (2) woman had complete medical information and has signed the informed consent form; (3) fetus had no known congenital malformation or chromosomal abnormality; (4) pregnant women without no medical disease.

#### Evaluation and analysis of ultrasound reports

All the ultrasound report analysis results were evaluated by four radiologists: two with two years of experience in obstetric ultrasound(X.J.L., W.M.Y.) and two with ten years of experience in obstetric ultrasound(D.Y.R., X.S.J.). Discrepancies in assessment among the two radiologists were resolved by a third ultrasound expert (J.Q.Z.) with more than twenty years of experience in obstetric ultrasonic diagnosis. Based on the ultrasound diagnostic results in these reports, we categorized them into reports with abnormal fetal growth measurements, abnormal amniotic fluid index, abnormal placental findings, and reports with normal results. Then, each ultrasound report was posed three times to the online ChatGPT-3.5, ChatGPT-4.0 (chat.openai.com) and Microsoft Copilot in Bing (bing.com) at different times, and their analysis of the ultrasound report and suggestions provided were recorded and compared. If a report contained more than one abnormality, each would be evaluated separately. The reviewers divided each set of responses into “consistent” or “inconsistent” based on the agreement among three responses, regardless of whether the conveyed concept was correct or incorrect. According to the accuracy of the report analysis, the results can be categorized into: (1) Accurate: Abnormal indicator in the report has been identified; (2) Inaccurate: Abnormal indicator was not detected, or normal ultrasound reports were mistakenly labeled as abnormal. According to the comprehensiveness of the suggestions provided, they can be classified as comprehensive or incomplete.

### Statistics

The proportions of responses for each grade were calculated. Statistical analysis was conducted using SPSS version 20.0 software (SPSS Inc., Chicago, IL, USA). A pairwise comparative analysis of the qualitative descriptions (consistency and accuracy) of the responses generated by ChatGPT-3.5, ChatGPT-4.0 and Copilot was performed using the Chi-square test for calibration. The *P*-values for each index were computed, with values < 0.05 considered statistically significant.

## Electronic supplementary material

Below is the link to the electronic supplementary material.

Supplementary Material 1.

Supplementary Material 2.

## Data Availability

Data is provided within the manuscript or supplementary information files.
