# Supplementary material for: Performance of ChatGPT and Microsoft Copilot in Bing in answering obstetric ultrasound questions and analyzing obstetric ultrasound reports
Source: Sci Rep. 2025 Apr 26;15:14627. doi: 10.1038/s41598-025-99268-2 (PMC12033324; doi:10.1038/s41598-025-99268-2)
Supplement: Supplementary file 1 — Supplementary Material 1. [file 41598_2025_99268_MOESM1_ESM.docx]

**Supplementary Material**

**Table S1.** **An example of some correct and some incorrect responses generated by Microsoft Copilot in Bing**

| Questions | | How many times and how often do the obstetric ultrasound examinations should be done during pregnancy? |
| --- | --- | --- |
| Responses | Trial 1 | The number and frequency of obstetric ultrasound examinations depend on your pregnancy condition and your health care provider’s recommendations. A healthy pregnancy typically requires two ultrasounds: one around 11 to 14 weeks and the other at about 18 to 20 weeks. If any abnormalities or complications are detected during either of the routine ultrasounds, you might need more. In high-risk pregnancies - usually due to the weight, age, or medical history- weekly ultrasounds may be recommended during the final month of pregnancy. Some health care providers may also use ultrasounds in the first trimester to confirm pregnancy, establish gestational age, determine the number of fetuses, or diagnose an ectopic pregnancy or miscarriage. |
|  | Trial 2 | The number of obstetric ultrasound examinations during pregnancy varies depending on the pregnancy care provider and if you have any health conditions. Most pregnant people have one or two ultrasounds during pregnancy. [However, if your pregnancy is high risk or if your provider suspects you or your baby has a health condition, they may suggest more frequent ultrasounds](https://my.clevelandclinic.org/health/diagnostics/9704-ultrasound-in-pregnancy). |
|  | Trial 3 | Most healthy women receive two ultrasound scans during pregnancy. [The first is ideally in the first trimester to confirm the due date, and the second is at 18-22 weeks to confirm normal anatomy and the sex of the baby](https://www.bidmc.org/about-bidmc/wellness-insights/pregnancy/2018/09/ultrasounds-during-pregnancy-how-many-and-how-often). [However, the number and timing of ultrasounds vary depending on your pregnancy care provider and if you have any health conditions](https://my.clevelandclinic.org/health/diagnostics/9704-ultrasound-in-pregnancy). |
| Why responses were some correct and some incorrect? | | ISUOG practice guidelines^[25]^ suggests that the fetal growth assessment usually requires at least two ultrasound scans performed at separate time points: one before 14 weeks and another after 14 weeks. Due to various factors such as age, weight, and medical history, additional ultrasounds may be needed. The response in trial 2 to the question of conducting just one ultrasound during pregnancy may be incorrect. |

ISUOG : International Society of Ultrasound in Obstetrics and Gynecology

**Table S2.** **An example of some correct and some incorrect responses generated by ChatGPT-3.5**

| Questions | | What is the amniotic fluid index? What is the normal value of amniotic fluid index? |
| --- | --- | --- |
| Responses | Trial 1 | The AFI is a measurement used to assess the amount of amniotic fluid surrounding the fetus during pregnancy. It is determined by ultrasound examination and provides an estimate of the quantity of amniotic fluid in the amniotic sac.  The AFI is calculated by dividing the uterus into four quadrants and measuring the deepest vertical pocket of amniotic fluid in each quadrant. The measurements from the four quadrants are added together to obtain the AFI value.  The normal range for the Amniotic Fluid Index is typically considered to be between 5 and 25 centimeters (cm). This range may vary slightly depending on the gestational age. However, it's important to note that the interpretation of AFI values should be done in conjunction with other clinical factors, such as gestational age, fetal well-being, and maternal conditions.  An AFI value within the normal range suggests that there is an adequate amount of amniotic fluid to support the baby's growth and development. Amniotic fluid plays crucial roles in protecting the fetus, allowing movement and development of the musculoskeletal system, regulating temperature, and promoting lung development.  Abnormal AFI values can indicate conditions such as oligohydramnios (too little amniotic fluid) or polyhydramnios (excessive amniotic fluid). Oligohydramnios can be associated with fetal growth restriction, placental insufficiency, or fetal kidney problems, among other factors. Polyhydramnios may be linked to conditions like gestational diabetes, fetal anomalies, or fetal swallowing difficulties.  If an AFI measurement falls outside the normal range, further evaluation and monitoring may be necessary to determine the cause and manage the condition appropriately. Healthcare providers will consider the overall clinical picture, conduct additional tests if needed, and make decisions based on the best interests of both the mother and the baby.  It's important to consult with your healthcare provider for any concerns or questions regarding the amniotic fluid index and its implications for your pregnancy. They have the expertise to interpret the ultrasound findings and provide appropriate guidance based on your individual circumstances. |
|  | Trial 2 | The AFI is a measurement used to assess the amount of amniotic fluid surrounding the fetus during pregnancy. It is a quantitative method to evaluate the amniotic fluid volume and is typically measured during an obstetric ultrasound examination.  The AFI is determined by dividing the uterus into four quadrants and measuring the deepest vertical pocket of amniotic fluid in each quadrant. The measurements from each quadrant are then summed to obtain the AFI value.  The normal value for the Amniotic Fluid Index is typically considered to be between 5 and 25 centimeters (or sometimes stated as 5 to 24 centimeters). This range may slightly vary depending on the healthcare provider or institution.  The amniotic fluid serves several important functions during pregnancy, including cushioning and protecting the fetus, allowing for fetal movement, aiding in lung development, and providing a stable environment for fetal growth. The AFI measurement helps assess the adequacy of amniotic fluid volume and can provide information about fetal well-being.  If the AFI falls below the lower end of the normal range (less than 5 centimeters), it is referred to as oligohydramnios, which indicates a low amniotic fluid volume. Oligohydramnios may be associated with various factors, such as fetal growth restriction, placental dysfunction, certain maternal conditions, or ruptured membranes.  On the other hand, if the AFI is above the upper end of the normal range (greater than 25 centimeters), it is referred to as polyhydramnios, which indicates an excessive amniotic fluid volume. Polyhydramnios can be associated with conditions such as gestational diabetes, fetal abnormalities, multiple gestations (twins or more), or maternal conditions like diabetes or Rh sensitization.  When the AFI deviates from the normal range, further evaluation may be needed to determine the underlying cause and to assess the well-being of the fetus. Additional tests or follow-up ultrasound examinations may be conducted to monitor the amniotic fluid levels and ensure appropriate management of the pregnancy.  It's important to consult with your healthcare provider regarding any concerns or questions about the amniotic fluid index or its implications in your specific pregnancy. They can provide personalized information and guidance based on your individual circumstances and medical history. |
|  | Trial 3 | The AFI is a measurement used to assess the amount of amniotic fluid surrounding the fetus during pregnancy. It is a component of the biophysical profile, which is a prenatal assessment of fetal well-being.  The AFI is determined by using ultrasound to measure the deepest vertical pockets of amniotic fluid in four different quadrants of the uterus. The measurements are then summed together to obtain the AFI value.  The normal value for the AFI is typically considered to be between 8 and 18 centimeters. However, it's important to note that the specific range may vary slightly depending on the gestational age of the fetus and the healthcare provider's preferences.  A lower AFI value, below the normal range, may indicate decreased amniotic fluid levels, a condition known as oligohydramnios. Oligohydramnios can be associated with various complications, including fetal growth restriction, placental problems, kidney issues in the fetus, or rupture of membranes (premature rupture of membranes). Close monitoring and further evaluation may be required to determine the cause and manage oligohydramnios appropriately.  On the other hand, a higher AFI value, above the normal range, may suggest an excessive amount of amniotic fluid, a condition called polyhydramnios. Polyhydramnios can be associated with conditions such as gestational diabetes, fetal abnormalities, multiple pregnancies (twins, triplets), or genetic disorders. Similar to oligohydramnios, further investigation and management may be necessary to address the underlying cause and monitor the well-being of both the mother and the fetus.  It's important to keep in mind that the AFI is just one component of the overall assessment of fetal well-being, and the interpretation of the results should be done in consultation with a healthcare provider. They will take into account other factors and clinical considerations to determine the appropriate course of action. |
| Why responses were some correct and some incorrect? | | Ultrasound scans measure amniotic fluid levels using the AFI; normal levels range from 5 to 25 cm^[28]^. The response to the normal amniotic fluid index range of 8-18cm in trial 3 may be incorrect. |

AFI: Amniotic Fluid Index

**Table S3.** **An example of some correct and some incorrect responses generated by ChatGPT-4.0**

| Questions |  | What does placental maturity level II mean? |
| --- | --- | --- |
| Responses | Trial 1 | Placental maturity level II refers to a mid-maturity stage of the placenta, which is typically observed in the third trimester of pregnancy, but it can also appear toward the end of the second trimester. It represents a transitional stage between an immature placenta (level I) and a mature placenta (level III).  Characteristics of Placental Maturity Level II:  Appearance: The placenta shows some scattered calcifications and mild indentations on its surface. These changes indicate the early signs of placental aging but are not yet extensive. The placenta may have small, shallow lobulations or slight indentations (areas of slight thinning or depressions) that form in the placental tissue. The overall structure remains smooth, but it is less uniform than level I.  Gestational Age: Level II maturity is typically seen during the late second trimester and early third trimester, around 25 to 32 weeks gestation. At this stage, the placenta is still relatively healthy and efficient in performing its functions, but signs of maturation (such as mild calcification) are beginning to appear.  Function: The placenta at level II maturity is still functioning well and capable of providing adequate oxygen and nutrients to the fetus. While the placenta may show signs of beginning to mature, it is still considered healthy and there is generally no indication of placental insufficiency at this stage.  Significance of Placental Maturity Level II:  Transition Point: This stage indicates that the placenta is transitioning from the immature phase (level I) to the more mature phase (level III) as the pregnancy progresses.  Fetal Growth and Development: Placental maturity level II is considered normal during this period of pregnancy and does not typically indicate any problems. However, the appearance of slight calcifications or indentations means that the placenta is undergoing the normal aging process as it prepares for delivery.  Ultrasound Interpretation: When a healthcare provider sees placental maturity level II, it is generally reassuring and suggests that the pregnancy is progressing well. However, if significant changes or more advanced calcifications are observed in the placenta (such as level III or IV maturity), this might require further investigation.  Comparison to Other Placental Maturity Levels:  Level I (Immature Placenta): Appears smooth and uniform, without calcifications, and is typically seen in the early second trimester.  Level II (Mid-Mature Placenta): Shows mild calcifications and slight indentations, typical for the late second to early third trimester.  Level III (Mature Placenta): Shows prominent calcifications and lobulations, often seen in the third trimester after 34 weeks.  Level IV (Old Placenta): Exhibits extensive calcifications and significant areas of necrosis, indicating a very aged placenta. This can be a concern if it occurs prematurely (before 37 weeks), as it may indicate placental insufficiency.  Clinical Significance of Placental Maturity Level II:  No Immediate Concern: A placental maturity level II generally does not pose a risk to the pregnancy, as the placenta is still functioning well.  Monitoring: While placental level II is considered normal in the late second and early third trimester, it is important to continue monitoring the placenta and fetal growth during later ultrasounds to ensure that placental maturation continues appropriately and to rule out any issues with placental insufficiency.  Fetal Development: The placenta at this stage is still well-formed and adequately supplying the fetus with nutrients and oxygen, contributing to healthy fetal growth.  Conclusion: Placental maturity level II signifies a mid-maturity stage of the placenta and is usually observed around 25 to 32 weeks of gestation. It is a normal and expected stage of placental development, showing mild signs of aging, such as scattered calcifications and indentations. At this stage, the placenta is still functional and supports fetal growth effectively. It is important to continue monitoring the placenta in later stages of pregnancy to ensure that it remains healthy and provides optimal support for fetal development. |
|  | Trial 2 | Placental maturity level II refers to a stage of development of the placenta that is typically observed in the late third trimester of pregnancy, usually between 34 and 38 weeks of gestation. This stage indicates that the placenta is becoming more mature and showing more significant changes compared to earlier stages. It is often characterized by calcifications and indentations that are more prominent than in Grade I (earlier maturity), but not as advanced as in Grade III (post-term maturity).  Placental Maturity Grading System: To better understand Grade II, here is a quick overview of the placental maturity grading system:  Grade 0: The placenta is immature, smooth, and homogeneous, seen in early pregnancy (before 28 weeks).  Grade I: This is the early stage of placental maturity, typically observed between 28 and 34 weeks, with small calcifications and minor indentations at the edges of the placenta.  Grade II: The placenta becomes more mature, seen between 34 and 38 weeks. There are more pronounced calcifications, indentations, and lobulations (small divisions within the placenta). The texture of the placenta may be uneven at this stage.  Grade III: The placenta is fully mature, typically seen after 38 weeks, or in post-term pregnancies. It may show deep calcifications, lobulations, and areas of infarction or reduced thickness, indicating aging and potential decreased efficiency in nutrient and oxygen transfer to the fetus.  What Does "Placental Maturity Level II" Mean?  Grade II represents a late third-trimester placenta, typically between 34-38 weeks. At this stage, the placenta is becoming more mature, showing increased calcifications, lobulations, and more indented areas along the placental surface. These changes are a normal part of the aging process and generally indicate that the placenta is still functioning adequately but is closer to the end of its useful life in the pregnancy.  Clinical Significance of Placental Maturity Level II:  Normal for Late Third Trimester: Grade II is typically normal in the late third trimester and suggests that the placenta is aging in a way that is expected as the pregnancy progresses toward full term. The placenta continues to function and provide nutrients and oxygen to the fetus at this stage, though it is maturing and preparing for delivery.  Possible Indicator of Age-Related Changes: While Grade II is generally normal for this stage, it is important to note that as the placenta matures, its ability to function may decline slightly. This could lead to a reduction in blood flow or nutrient transfer to the fetus. In some cases, an overly mature placenta (e.g., Grade III) before 38 weeks can indicate potential problems like placental insufficiency, where the placenta may not be providing enough oxygen or nutrients to the baby.  Monitoring in High-Risk Pregnancies: For women with risk factors like high blood pressure, diabetes, or previous pregnancy complications, a Grade II placenta may be monitored closely to ensure that it continues to function properly and does not show signs of insufficiency as the pregnancy nears its end. Doppler ultrasound can be used to check blood flow through the umbilical artery and other vessels to assess how well the placenta is functioning.  Preparation for Delivery: Grade II can also indicate that the body is preparing for delivery. The placenta may continue to age and mature toward a Grade III in the weeks leading up to full-term delivery (around 40 weeks).  Summary: Placental maturity level II occurs between 34 and 38 weeks of pregnancy and is generally considered normal in the late third trimester. It shows pronounced calcifications, indentations, and lobulations on the placental surface as part of the normal aging process. Grade II indicates that the placenta is still functioning well and providing nutrients and oxygen to the fetus, but is beginning to show signs of aging as the pregnancy nears its conclusion. While Grade II is typically not concerning on its own, careful monitoring may be required in certain high-risk pregnancies to ensure the placenta is working efficiently as it continues to mature. If you have concerns about placental function or maturity, your healthcare provider will likely monitor the pregnancy closely and may recommend additional assessments like Doppler ultrasound to ensure the placenta is adequately supporting the fetus. |
|  | Trial 3 | Placental maturity level II refers to a stage in the development of the placenta that typically occurs during the late second trimester to early third trimester (around 24-34 weeks of gestation). At this stage, the placenta has undergone some maturation compared to level I but is still considered normal for the gestational age, assuming there are no additional complications.  What Does Placental Maturity Level II Mean?  What it indicates: A level II placenta is considered intermediate in maturity. It reflects normal changes occurring as the placenta ages, but these changes are generally still physiological and not necessarily concerning. This stage suggests that the placenta is developing further but hasn't reached the level of mature or aged placenta (grade III), which is expected closer to term.  Ultrasound Appearance: Small calcifications may appear in the maternal surface (the side of the placenta facing the uterus). The chorionic plate (outer surface) might show some slight indentations. There may be minor fissures or slight areas of separation within the placenta. These are generally mild and do not typically affect its function. The placenta may have a less uniform appearance compared to level I, with some hyperechoic areas (brighter spots) due to early calcifications or aging.  Placental Function: While there are early signs of aging, the placenta is still functioning well and is typically sufficient for the needs of the fetus. It is still providing oxygen and nutrients adequately. It is important to note that placental insufficiency is not typically associated with level II maturity, as this is still within the normal range for pregnancy at this stage.  Gestational Age: A level II placenta is typically seen in pregnancies between 24 and 34 weeks of gestation. This is a normal part of the aging process of the placenta as the pregnancy progresses.  How Placental Maturity Progresses: Placental maturity moves through a series of stages as the pregnancy continues.  Here is how the process typically looks:  Grade I (Immature placenta): This is the early stage, typically found during 18- 24 weeks. Placenta appears smooth, without calcifications or significant aging.  Grade II (Intermediate maturity): This occurs in the late second trimester to early third trimester (24 -34 weeks). The placenta shows small calcifications, indentations, and minor fissures, but remains functional and healthy.  Grade III (Mature placenta): This is seen closer to term (typically after 34 weeks of gestation), and the placenta shows prominent calcifications, larger indentations, and more visible aging changes. While this is normal for the third trimester, if it appears too early, it can signal placental insufficiency or other complications.  Grade IV (Advanced maturity): This is less commonly used but refers to an advanced stage of placental aging, often seen in cases of early placental aging or placental insufficiency.  Clinical Significance of Placental Maturity Level II: A level II placenta is generally normal and expected for pregnancies in the late second to early third trimester. However, it does indicate that the placenta is beginning to mature, and this can have clinical significance for the following reasons:  Fetal Monitoring: While placental maturity level II is typically normal for the gestational age, it signals the need for regular monitoring as the pregnancy progresses. The presence of early calcifications or minor fissures might prompt healthcare providers to monitor fetal well-being and growth, particularly if there are concerns about placental insufficiency or IUGR (intrauterine growth restriction).  Placental Function: If the placenta matures too early (e.g., grade III or IV before 34 weeks), it may indicate placental insufficiency or poor function. However, level II by itself does not generally suggest such issues. Minor calcifications and indentations are common in normal pregnancies and do not typically impact placental function unless they are severe.  Preterm Birth Risk: In some cases, premature placental aging (e.g., early grade III) can be associated with an increased risk of preterm birth or growth restriction. However, level II is still well within the normal range for gestational age.  Conclusion: Placental maturity level II represents a normal stage of placental development that typically occurs in the late second trimester to early third trimester (around 24 -34 weeks). At this stage, the placenta may show signs of early aging, such as minor calcifications, indentations, and slight fissures, but it is still considered to be functioning well and providing the necessary nutrients and oxygen to the fetus. A level II placenta is generally not concerning, but as with all stages of placental maturity, it is important for healthcare providers to continue monitoring the pregnancy to ensure that the placenta continues to function appropriately as the pregnancy progresses toward full term. |
| Why responses were some correct and some incorrect? | | Placental maturity level II refers to a stage in the development of the placenta that typically occurs during the late second trimester to early third trimester (around 24-34 weeks of gestation) ^[29]^. The response in Trial 2 to the late third trimester of pregnancy (34-38 weeks of gestation) may be incorrect. |

**Table S4. Example of ultrasound report analysis results**

| Accurate ultrasound analysis report | Ultrasound report | A 32-year-old pregnant woman underwent an ultrasound examination at 35 weeks and 5 days of pregnancy. The examination showed the following:  Number of fetuses: 1. Fetal position: cephalic. Fetal heart rate: visible. Growth parameters: Biparietal diameter: 90 mm; Head circumference: 318 mm; Abdominal circumference: 312 mm; Femur length: 66 mm; Humerus length: 56 mm. Placental position: posterior wall; Placental thickness: 36 mm; Placental maturity: Grade II+, the placenta completely covers the cervical opening. Amniotic fluid index: 41-37-0-40 mm.  Color Doppler imaging: Color blood flow visible in the heart cavity; Umbilical artery: PI: 0.75, RI: 0.53, S/D: 2.13, FHR: 155 bpm. Could you please identify which indicator is abnormal? If there are any abnormalities, what suggestions do you have? |
| --- | --- | --- |
|  | Analysis report by ChatGPT -3.5 | Based on the provided ultrasound examination results, the following indicators are abnormal:  Placental Edge Location: The description "The distance between the lower edge of the placenta and the cervical opening completely covers the cervical opening" indicates placenta previa, a condition where the placenta partially or completely covers the cervix. This is abnormal and can pose risks during labor and delivery.  AFI: The AFI value of 0mm in one quadrant suggests severe oligohydramnios in that area, which is abnormal. Oligohydramnios can affect fetal well-being and can be associated with various maternal and fetal conditions.  Suggestions if abnormalities are detected:  Placenta Previa: Placenta previa requires careful monitoring and management, as it increases the risk of bleeding during labor and delivery. Depending on the severity and other factors, the healthcare provider may recommend bed rest, avoiding intercourse, and planning for a cesarean delivery to minimize the risk of bleeding.  Oligohydramnios: Severe oligohydramnios requires prompt evaluation and management to assess fetal well-being and determine the underlying cause. The healthcare provider may recommend additional testing, such as non-stress tests or biophysical profiles, to monitor fetal health. In some cases, interventions such as amnioinfusion or early delivery may be necessary to prevent complications associated with oligohydramnios.  It's crucial for the pregnant woman to discuss these findings with her healthcare provider promptly for further evaluation and appropriate management, as these conditions can have significant implications for both the mother and the baby. |
|  | Analysis report by ChatGPT -4.0 | Let's review the ultrasound report and assess the potential abnormalities: 1. Fetal Position (Head Position) Observation: The fetus is in the head-down (cephalic) position, which is ideal for delivery. Conclusion: Normal. No concern here.  2. Placental Location (Placenta Previa) Observation: The lower edge of the placenta is described as completely covering the cervical opening, indicating placenta previa. Potential Issue: Placenta previa is a condition where the placenta is positioned over or near the cervix, which can obstruct the birth canal. This can cause bleeding during pregnancy and complicate vaginal delivery. Suggestion: Placenta previa requires close monitoring. Depending on the severity of the previa and whether the placenta migrates as the pregnancy progresses, a cesarean section may be necessary. If bleeding occurs or the placenta does not move away from the cervix, a C-section will likely be recommended at the time of delivery.  3. Placental Thickness (36mm) and Maturity (Grade II+) Observation: The placenta is 36mm thick and Grade II+ in terms of maturity. Conclusion: A Grade II+ placenta at 35 weeks is within the normal range, though it indicates moderate placental aging. Grade II+ is considered a "moderately mature" placenta, which can still function normally, but there may be reduced efficiency in nutrient and oxygen transfer as pregnancy advances. Suggestion: Normal for this stage of pregnancy, but continuous monitoring of fetal growth may be recommended to ensure the placenta is still functioning well.  4. Amniotic Fluid Index (AFI) Observation: T The AFI is 41-37-0-40mm, totaling 118mm, which is on the higher side but still within the normal range. Potential Issue: Although the AFI is higher than average, it does not meet the threshold for polyhydramnios, which is typically defined as an AFI greater than 25cm. This value indicates slightly increased amniotic fluid, but it is still within normal limits. Suggestion: While the fluid level is slightly higher, there is no significant abnormality at this point. Continued monitoring of the amniotic fluid and further evaluation may be required as the pregnancy progresses.  5. Umbilical Artery Doppler (PI = 0.75, RI = 0.53, S/D = 2.13) Observation: The Doppler indices for the umbilical artery are: PI (Pulsatility Index) = 0.75 (normal is typically <1.0) RI (Resistive Index) = 0.53 (normal is typically between 0.50 and 0.70) S/D Ratio (Systolic/Diastolic Ratio) = 2.13 (normal is typically <3.0) Conclusion: These values are within normal ranges, indicating that blood flow in the umbilical artery is normal and there is no sign of placental insufficiency. Conclusion: Normal.  6. Fetal Heart Rate (FHR = 155 bpm) Observation: The fetal heart rate is 155 bpm, which is within the normal range (typically 110–160 bpm). Conclusion: Normal. The fetal heart rate indicates no immediate signs of distress.  Summary of Potential Abnormalities: The placenta is completely covering the cervix, which may interfere with a vaginal delivery. This is the primary concern.  Suggestions: This condition will need to be monitored closely. The placenta may migrate as the uterus grows, but if it continues to cover the cervix, a cesarean section will be planned to avoid complications during vaginal delivery. Regular ultrasounds and consultations with your obstetrician are essential.  Be sure to follow up with your healthcare provider for detailed management based on these findings, particularly for placenta previa. |
|  | Analysis report by Microsoft Copilot in Bing | Certainly! Let’s analyze the ultrasound examination findings for any potential abnormalities:  Fetal Measurements:  Biparietal Diameter: 90 mm  Head Circumference: 318 mm  Abdominal Circumference: 312 mm  Femoral Length: 66 mm  Humeral Length: 56 mm  Placental Details:  Placental Orientation: Posterior wall  Placental Thickness: 36 mm  Placental Maturity: Grade II+  Placental Location: The lower edge of the placenta completely covers the cervical opening.  Amniotic Fluid Measurements:  AFI:  Maximum vertical pocket: 41 mm  Minimum vertical pocket: 0 mm  Color Doppler Imaging:  Color blood flow is visible in the heart cavity.  Umbilical Artery Doppler Indices:  PI: 0.75  RI: 0.53  S/D: 2.13  FHR: 155 bpm  Now, let’s address any potential abnormalities:  AFI: The AFI values show fluctuations, with a maximum pocket of 41 mm and a minimum pocket of 0 mm. While some variation is normal, consult your healthcare provider to ensure adequate amniotic fluid levels.  Placental Location: The complete coverage of the cervical opening by the placenta may impact the mode of delivery. Discuss this finding with your obstetrician to plan accordingly.  Fetal Measurements: The fetal measurements appear within the normal range, but individual variations exist. Regular monitoring is essential to ensure continued growth and well-being.  Remember that personalized advice depends on your specific health condition, and your healthcare provider will guide you based on these findings. Regular prenatal check-ups are crucial during this stage of pregnancy. |
| Inaccurate ultrasound analysis report | Ultrasound report | A 28-year-old pregnant woman underwent an ultrasound examination at 32 weeks and 0 days of pregnancy. The examination results were as follows:  Number of fetuses: 1.Fetal position: cephalic. Fetal heart rate: visible. Growth parameters: Biparietal diameter: 76 mm; Head circumference: 258 mm; Abdominal circumference: 251 mm; Femur length: 49 mm; Humerus length: 44 mm. Placental position: right wall; Placental thickness: 42 mm. Placental maturity: Grade II+; The distance between the lower edge of the placenta and the cervical opening is greater than 2 centimeters. Amniotic fluid index: 21-27-41-16 mm. Color Doppler imaging: Color blood flow visible in the heart cavity; Umbilical artery: PI: 2.00, RI: 1.00, FHR: 142 bpm; U-shaped incision seen in the neck. Are there any abnormal indicators? If there are any abnormalities, what suggestions do you have? |
|  | Analysis report by ChatGPT-3.5 | Based on the provided ultrasound examination results, there are several indicators that may be considered abnormal or concerning:  Placental Thickness and Maturity: While a placental thickness of 42mm and maturity grade II+ aren't necessarily abnormal on their own, they may indicate placental hypertrophy or other placental abnormalities that warrant close monitoring.  Distance Between Placenta and Cervical Opening: The distance between the lower edge of the placenta being greater than 2 centimeters is normal and suggests no placenta previa, which is reassuring.  AFI: The AFI values show significant variability, ranging from 16mm to 41mm. Such fluctuations may indicate issues with amniotic fluid dynamics, possibly indicating either oligohydramnios or polyhydramnios.  U-shaped Incision on the Neck: This finding, a U-shaped incision in the fetal neck (nuchal cord), is concerning as it suggests the umbilical cord is wrapped around the baby's neck. While this is relatively common and may not always lead to complications, it requires close monitoring.  Given these findings, the obstetrician should closely monitor the pregnancy and fetal well-being. Further evaluation may include additional ultrasounds, non-stress tests, and possibly consultation with a maternal-fetal medicine specialist. Closer monitoring of the placenta, amniotic fluid levels, and fetal growth may be warranted, especially due to the variability in the AFI. Depending on the specific circumstances, early delivery may be considered if there are concerns about fetal distress or compromised blood flow. Management of the nuchal cord during labor and delivery should also be discussed to minimize risks to the baby. |
|  | Analysis report by ChatGPT-4.0 | Analysis of the Ultrasound Results  Fetal Position: Head position-Normal for a 32-week pregnancy.  Fetal Heart Rate (FHR): 142 bpm- Normal. The fetal heart rate typically ranges from 110-160 bpm at this stage, so 142 bpm is within the normal range.  Growth Measurements:  Biparietal diameter (BPD): 76mm-This is within normal range for 32 weeks of pregnancy. Head circumference (HC): 258mm-This is also within normal range for 32 weeks. Abdominal circumference (AC): 251mm-This is within the expected range for 32 weeks. Femoral length: 49mm-This is normal for 32 weeks. Humeral length: 44mm-This is also normal for this gestational age.  Placental Orientation: Right wall-Normal. The placenta can be located in different positions in the uterus, and a right-wall position is a typical location.  Placental Thickness: 42mm-This is within the normal range for placental thickness at 32 weeks. The thickness typically varies from 20mm to 50mm at this stage of pregnancy.  Placental Maturity: Grade II+-This is within normal range for 32 weeks. Grade II or II+ maturity is expected for this gestational age, indicating that the placenta is functioning normally at this stage.  Distance Between Placenta and Cervical Opening: Greater than 2 cm- Normal. The distance between the placenta and the cervical opening should ideally be greater than 2 cm, which ensures there is no placenta previa.  Amniotic Fluid Index (AFI): 21-27-41-16mm, AFI = 105mm-This is within the normal range. Normal AFI at 32 weeks typically ranges from 80-250mm, and an AFI of 105mm is considered normal.  Umbilical Artery Doppler:  PI (Pulsatility Index): 2.00-Normal. A PI of 2.00 is within the expected range for a 32-week pregnancy. RI (Resistive Index): 1.00-Normal. An RI of 1.00 is also within the expected range at this stage. Fetal Heart Rate (FHR): 142 bpm-Normal.  Potential Concerns and Suggestions  Umbilical Artery Doppler (PI and RI):  The Pulsatility Index (PI) of 2.00 and the Resistive Index (RI) of 1.00 fall within normal ranges, suggesting adequate blood flow through the umbilical artery and good placental function at this time.  No significant concerns from Doppler study in this case.  Placental Maturity (Grade II+):Grade II+ placental maturity is typical for 32 weeks of gestation. It indicates that the placenta is developing as expected. Placental aging begins to be seen in later weeks of pregnancy, but there are no signs of significant dysfunction at this stage.  Amniotic Fluid Index (AFI): The AFI = 105mm is well within the normal range, so there are no concerns about the amount of amniotic fluid.  Overall Conclusion:  The ultrasound results are normal, with all the key measurements and Doppler results falling within expected ranges for a 32-week pregnancy.  Suggestions for Follow-Up:  Monitor Fetal Movement: Ensure the mother continues to monitor fetal movement. If there is any noticeable decrease in movement, she should contact her healthcare provider promptly.  Routine Prenatal Visits: Continue with regular prenatal visits and ultrasound monitoring as recommended by the doctor to track fetal growth and well-being, as well as maternal health.  Consider Fetal Monitoring: If the patient has any concerns about the pregnancy (such as discomfort or a history of complications), non-stress tests (NST) or a biophysical profile (BPP) could be considered for further monitoring.  Prepare for Delivery: As the pregnancy is nearing full-term, the mother should begin to prepare for labor and delivery, and ensure she has discussed her birth plan with her healthcare provider.  Discuss Placental Position: The placenta is positioned on the right wall and is not near the cervix. Continue monitoring to ensure the placenta remains away from the cervical opening.  As of now, there is no indication of abnormality in the ultrasound results. The pregnancy appears to be progressing well, with normal fetal development and placental function. |
|  | Analysis report by Microsoft Copilot in Bing | The ultrasound examination results you’ve provided indicate several measurements related to fetal growth and well-being. Let’s break down each parameter and discuss their significance:  Biparietal Diameter (BPD): The BPD measures the widest part of the fetal head. At 32 weeks, a BPD of 76mm is within the normal range. It helps assess fetal growth and development.  Head Circumference (HC): The HC reflects the size of the fetal skull. A measurement of 258mm is generally normal at this stage of pregnancy.  Abdominal Circumference (AC): AC measures the circumference of the fetal abdomen. A value of 251mm falls within the expected range for 32 weeks.  Femoral Length (FL): FL indicates the length of the fetal thigh bone. A measurement of 49mm is appropriate for this gestational age.  Humeral Length (HL): HL represents the length of the fetal upper arm bone. A value of 44mm is consistent with normal growth.  Placental Orientation and Thickness: The right wall orientation is typical for the placenta. A thickness of 42mm is within the acceptable range. However, the distance between the lower edge of the placenta being greater than 2 centimeters may require further evaluation.  Placental Maturity: Grade II+ suggests that the placenta is functioning well but is showing some signs of aging. Regular monitoring is essential to ensure continued fetal well-being.  AFI: The AFI values (21-27-41-16mm) represent the amount of amniotic fluid around the baby. While fluctuations are normal, a sudden drop (from 41mm to 16mm) may warrant closer observation.  U-Shaped Incision in Fetal Neck: This finding requires attention. It could be related to fetal positioning or other factors. Consultation with a specialist is advisable to assess its significance.  Abnormal Indicators:  The decreased AFI at 32 weeks (16mm) is concerning. Low amniotic fluid levels can impact fetal development and may indicate issues such as placental insufficiency or fetal kidney problems.  The U-shaped incision in the fetal neck should be evaluated promptly to rule out any structural abnormalities.  Suggestions:  Follow-Up Ultrasound: Arrange for a follow-up ultrasound to monitor amniotic fluid levels and assess fetal growth. This will help determine if any interventions are necessary.  Consult a Maternal-Fetal Medicine Specialist: Seek advice from a specialist who can provide detailed insights based on the specific findings. They may recommend additional tests or interventions.  Monitor Fetal Movements: Pay attention to fetal movements. Reduced activity could be a sign of distress.  Stay Hydrated: Adequate hydration can help maintain amniotic fluid levels.  Discuss Delivery Options: Depending on the overall assessment, your healthcare provider may discuss the optimal timing for delivery.  Remember that each pregnancy is unique, and personalized guidance from your healthcare team is crucial. Regular prenatal visits and open communication with your doctor will ensure the best outcomes for both you and your baby. |
|  | Why was the ultrasound analysis report incorrect? | ChatGPT-4.0 suggests that the findings are entirely normal, while both ChatGPT-3.5 and Microsoft Copilot in Bing recommend monitoring the amniotic fluid and the U-shaped incision on the fetal neck, but they didn’t detect any abnormal indicators for the fetus. Specifically, the biparietal diameter, head circumference, abdominal circumference, femur length, and humerus length of the fetus were all less than -2 SD^[17,30]^. Three days after the ultrasound examination, the pregnant woman underwent a cesarean section due to premature labor. She was clinically diagnosed with a single live birth, delayed fetal growth and development, and a low birth weight infant. |

AFI: Amniotic Fluid Index; PI: Pulsatility Index; RI: Resistance Index; S/D : Systolic/ Diastolic Ratio; FHR: Fetal Heart Rate. BPD: Biparietal Diameter; HC : Head Circumference; AC: Abdominal Circumference; FL : Femoral Length; HL: Humeral Length; SD: Standard Deviation.

**Table S5.Twenty questions about obstetric ultrasound examination**

| No | Questions | ChatGPT-3.5 | | ChatGPT-4.0 | | Microsoft Copilot in Bing | |
| --- | --- | --- | --- | --- | --- | --- | --- |
|  |  | Consistency | Accuracy^*^ | Consistency | Accuracy^*^ | Consistency | Accuracy^*^ |
| 1 | What is obstetric ultrasound examination? | consistent | 1 | consistent | 1 | consistent | 2 |
| 2 | How many times and how often do the obstetric ultrasound examinations should be done during pregnancy? | consistent | 1 | consistent | 1 | inconsistent | 3 |
| 3 | Is there any effects on the fetus when obstetric ultrasound examinations were done very frequently? | consistent | 1 | consistent | 1 | consistent | 1 |
| 4 | Is there any effects on the fetus when transvaginal ultrasound examinations were done during pregnancy? Will it cause premature birth? | consistent | 1 | consistent | 1 | consistent | 1 |
| 5 | Can I eat food and drink water before obstetric ultrasound examination? | consistent | 2 | consistent | 1 | inconsistent | 2 |
| 6 | How can I read the measurements of fetal indicators on obstetric ultrasound examinations, such as biparietal diameter, head circumference, abdominal circumference, femur and humeral length? | consistent | 1 | inconsistent | 2 | consistent | 2 |
| 7 | What does it mean if the biparietal diameter/ head circumference/ abdominal circumference/ femur and humeral length is less than -2SD? | consistent | 2 | consistent | 1 | consistent | 2 |
| 8 | How to estimate fetal weight based on obstetric ultrasound measurements? | consistent | 1 | consistent | 1 | consistent | 1 |
| 9 | What is fetal orientation in obstetric ultrasound? | consistent | 1 | consistent | 1 | consistent | 2 |
| 10 | What does placental maturity level I mean? | consistent | 1 | consistent | 1 | inconsistent | 3 |
| 11 | What does placental maturity level II mean? | consistent | 1 | inconsistent | 3 | inconsistent | 3 |
| 12 | What do placental maturity level III, III_a_, III_b_ mean? | consistent | 1 | inconsistent | 2 | inconsistent | 3 |
| 13 | Why should the distance between the lower margin of the placenta and the internal cervical orifice orificium internum uteri be measured? What does it mean if this distance is less than 2cm? | inconsistent | 2 | consistent | 1 | consistent | 2 |
| 14 | What is the amniotic fluid index? What is the normal value of amniotic fluid index? | inconsistent | 3 | consistent | 2 | consistent | 1 |
| 15 | What causes and treatments of oligohydramnios or polyhydramnios? | consistent | 1 | consistent | 1 | consistent | 2 |
| 16 | What is the umbilical artery? What do the blood flow parameters PI, RI and S/D of umbilical artery respectively represent and how much is normal? | consistent | 1 | consistent | 1 | consistent | 1 |
| 17 | What should I do if obstetric ultrasound shows cord around neck? | consistent | 1 | consistent | 1 | consistent | 1 |
| 18 | What does high resistance index of umbilical artery mean? | consistent | 1 | consistent | 1 | consistent | 2 |
| 19 | What is the normal fetal heart rate? What do fetal heart rate tachycardia and bradycardia mean respectively? | consistent | 1 | consistent | 2 | consistent | 2 |
| 20 | Does the obstetric ultrasound report indicate that all the previous measurement indicators are normal, indicating that the fetus is completely normal? | consistent | 2 | consistent | 1 | consistent | 1 |

* The accuracy of each response was graded with the 4 scales: 1. Comprehensive: Defined as accurate and comprehensive, nothing more need be added by an experienced radiologist; 2. Correct but inadequate: All information is correct but incomplete; Some important information should be added by an experienced radiologist; 3. Some correct and some incorrect; 4. Completely incorrect.
